# A Terbinafine Sensitive *Trichophyton indotineae* Strain in Italy: The First Clinical Case of *tinea corporis* and onychomycosis

**DOI:** 10.3390/jof9090865

**Published:** 2023-08-22

**Authors:** Silvia Crotti, Deborah Cruciani, Sara Spina, Vincenzo Piscioneri, Ylenia Natalini, Giovanni Pezzotti, Michela Sabbatucci, Manuela Papini

**Affiliations:** 1 Centro Specialistico Patologie Micotiche, Istituto Zooprofilattico Sperimentale dell’Umbria e delle Marche “Togo Rosati” (IZSUM), 06126 Perugia, Italy; s.crotti@izsum.it (S.C.); s.spina@izsum.it (S.S.); v.piscioneri@izsum.it (V.P.); g.pezzotti@izsum.it (G.P.); 2 Clinica Dermatologica di Terni, Università degli Studi di Perugia, 06123 Perugia, Italy; ylenia.natalini@gmail.com (Y.N.); manuela.papini@unipg.it (M.P.); 3 Department of Infectious Diseases, Istituto Superiore di Sanità, 00161 Roma, Italy; michela.sabbatucci@iss.it; 4 Dipartimento di Medicina Clinica e Sperimentale, Università degli Studi di Pisa, 56126 Pisa, Italy

**Keywords:** EpiPulse, Italy, onychomycosis, *SQLE* gene, *tinea corporis*, *Trichophyton indotineae*

## Abstract

*Trichophyton indotineae* is an emerging dermatophyte species that plays a relevant role in human healthcare. It has been associated with severe chronic skin infections and a high level of terbinafine resistance. *T. indotineae* is endemic to India, Iran, and Iraq but several cases have been reported in Europe, recently. In this manuscript, the authors report the first clinical description of a *tinea corporis* and onychomycosis due to *T. indotineae*. The patient was a 42-year-old female from India that has lived in Umbria (Central Italy) for the last two years. Firstly, a dermatological examination suggested dermatophytosis: mycology isolation from cultures and macro- and microscopical features identified the colonies as belonging to the *T. mentagrophytes*/*T. interdigitale* species complex. Subsequently, ITS1/ITS4 end-point PCR and Sanger sequencing identified the strain as *T. indotineae*. Lastly, a DermaGenius^®^ Resistance Multiplex real-time PCR assay was carried out, targeting the mutations in the *SQLE* gene to establish terbinafine resistance or susceptibility of the strain. The melting curve observed was compatible with wild-type positive control, identifying the strain as *T. indotineae* terbinafine-sensitive. An oral terbinafine treatment was associated with a topical ciclopirox nail solution, resulting in remission in its clinical manifestation. On 3 July 2023, the local Prevention Service notified the case to the Ministry of Health that then reported the information at national and international levels.

## 1. Introduction

Dermatophytoses are the most common fungal infections, with a worldwide distribution and an estimated global prevalence of 20–25% [1]. The most frequent species causing human dermatophytoses are *T. rubrum*, *T. mentagrophytes*, *T. interdigitale*, and *T. tonsurans*. These fungal agents are associated with a broad spectrum of skin lesions (e.g., *tinea corporis*, *tinea capitis*, *tinea manuum*, *tinea pedis*, and *tinea cruris*) and onychomycosis. 

Recently, a new species of dermatophyte has been identified from India. It is morphologically indistinguishable from *T. mentagrophytes* but exhibits an anthropophilic instead of zoophilic transmission pattern and it has usually a high level of terbinafine resistance. Moreover, it differs from *T. mentagrophytes* and *T. interdigitale* by only a few single nucleotide polymorphisms (SNPs) in the internal transcribed spacer (ITS) region [2]. Based on these features and molecular analysis, it was called *Trichophyton indotineae* in 2020 and it is also known as *T. mentagrophytes* genotype VIII [3].

*Trichophyton indotineae* is considered endemic to India, with a percentage ranging from 82.6% to 94.0%, Iran and Iraq, with percentages of 19.7% and 69.2%, respectively. This mycete was also detected in Bahrain, Canada, Japan, Libya, Saudi Arabia, Syria, Thailand, and Yemen; sporadic cases have been also observed in Australia, Cambodia, Oman, and Vietnam [4]. With regard to European countries, *T. indotineae* has been reported in Belgium, Denmark, Estonia, Finland, France, Germany, Greece, and Switzerland [4]. More recently, four cases of terbinafine-resistant *T. indotineae* strains causing *tinea corporis* have been found in Northern Italy [5]. Apart from human cases *T. indotineae* can also infect animals as reported by Jabet et al. [6]. In particular, Tartor et al. [7] described two calf cases in Egypt, a dog case in India, and other cases in unspecified animals in Poland. 

The conventional diagnostic techniques may not be conclusive enough to differentiate *T. indotineae* from other *T. mentagrophytes/T. interdigitale* complex strains. Therefore, its molecular identification is decisive considering that it is often associated with terbinafine-resistance due to a nucleotide substitution in the squalene monooxygenase (*SQLE*) gene.

In this manuscript, the authors report the first clinical description of *tinea corporis* and onychomycosis caused by *T. indotineae* in a patient who immigrated from India to Central Italy two years ago. The diagnosis was obtained via isolation and macro- and microscopic observation of the strain. Molecular assays were carried out to confirm conventional data obtained. A DermaGenius^®^ Resistance Multiplex real-time polymerase chain reaction (PCR) assay was conducted to evaluate the terbinafine resistance or susceptibility of the strain testing *SQLE* gene mutants (Phe397Leu, Leu393Phe, Leu393Ser, Phe397Ile, and Phe397Val).

Considering the identity and the features of the isolated fungal strain together with its emerging role in the public health, and following the specific request from the Ministry of Health for notification of eventual cases sent to the Italian regions on 22 May 2023, the authors reported this case to the Ministry of Health on 3 July, which reported it through the European surveillance portal for infectious diseases (EpiPulse) on 21 July.

## 2. Case Description

**Figure 1:**
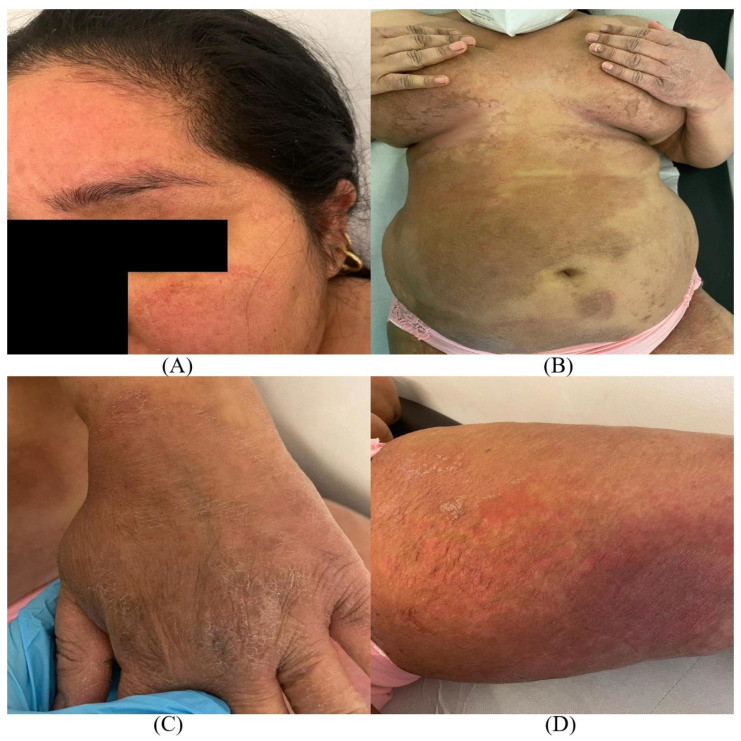
Widespread lesions of: (**A**) *tinea faciei*, (**B**) *tinea corporis*, (**C**) *tinea manuum*, and (**D**) *tinea cruris* characterized by red, scaly patches with sharp, slightly raised edges. The erythema was especially marked on the face and thighs.

A 42-year-old woman came to the Dermatologic Clinic of Santa Maria Hospital in Terni (Central Italy) showing *tinea corporis* with extensive lesions in association with onychomycosis. The patient, who immigrated from India to Italy two years ago, provided limited information about her medical history because of difficulties with the Italian language. Nevertheless, she reported the onset of cutaneous and nail lesions many months earlier, probably when she was still in India, and a previous treatment with topical ketoconazole and oral fluconazole that had not been effective. In detail, the patient reported that the first lesions were located on the groin and then spread rapidly to multiple sites. The dermatological examination revealed large, annular, scaly, and erythematous plaques with active red borders mainly located on the face, arms, trunk, groin, and legs. The lesions were also intensely pruritic and burning (Figure 1).

**Figure 2:**
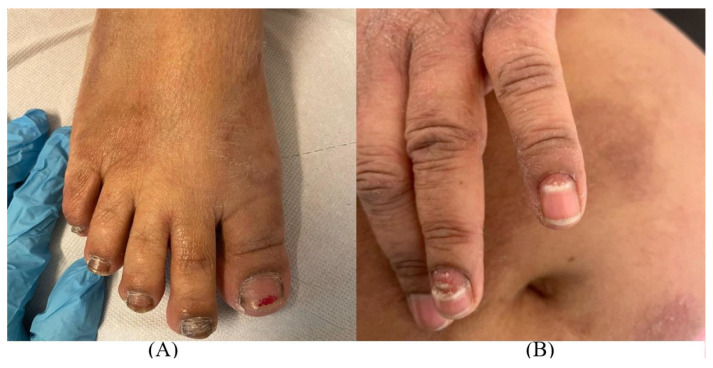
Lesions compatible with onychomycosis in (**A**) toenails and (**B**) fingernails.

Nails alterations were observed in toenails and fingernails. At the level of the second toe of the right foot, the nail plate appeared thickened and covered with friable whitish plaques in several points. Onychomadesis was present at the level of the fourth and fifth left finger with detachment, fragmentation, and fraying of the distal portion of the nail plate. All these aspects were compatible with the diagnosis of onychomycosis (Figure 2).

The patient reported that fortunately no one else in the family showed similar skin lesions. 

**Figure 3:**
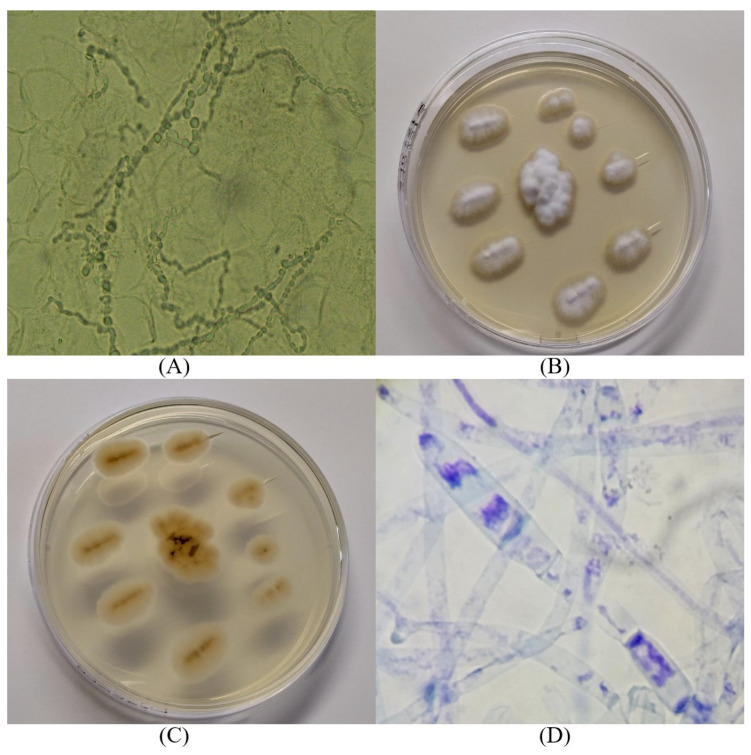
Conventional techniques for fungal diagnosis: (**A**) direct microscopic observation of the skin scraping samples; (**B**,**C**) macroscopic colonies morphology; (**D**) microscopic features of the colonies (methylene blue staining, 100×).

Skin specimens and proximal affected nails were sampled for fungal examination. To avoid contaminations, sampling was performed using a sterile scalpel blade to shave off scales from the borders of skin lesions and small pieces of nail keratin. The direct microscopic observation of the skin scraping samples showed septate hyphae and arthrospores (Figure 3A). The samples were cultured on Dermasel Agar (Sabouraud Agar by adding chloramphenicol and cycloheximide), incubated at 25 °C [8], and observed daily. Ten days later, a dermatophyte growth was observed. The colonies were flat, white, and had a macroscopic velvety aspect, with slight central elevation and jagged edges (Figure 3B). The reverse showed a light brown tint (Figure 3C). Microscopic features, performed through methylene blue staining, showed small and big round and oval microconidia and spindle-shaped septate macroconidia (Figure 3D).

Considering the medical history, dermatological examination, and the results of conventional techniques, a fungal infection due to *T. mentagrophytes*/*T. interdigitale* complex was hypothesized. Taking into account the severity and the extension of the skin and nails lesions, oral terbinafine 250 mg daily and topical ciclopirox nail solution were prescribed to be maintained for at least 12 weeks. Nevertheless, the geographical origin of the patient induced the authors to hypothesize a *T. indotineae* dermatophytosis. For this reason, at the same time, further laboratory investigations were performed.

To identify the fungal species, the isolated strain was conferred to the Specialist Centre for Fungal Diseases at the Istituto Zooprofilattico Sperimentale dell’Umbria e delle Marche “Togo Rosati” (IZSUM), where PCR and DNA sequencing were performed. The DNA was extracted using QIAamp DNA mini kit (QIAGEN^®^, Hilden, Germany) following a modified Gram-positive protocol (Appendix D: Protocols for Bacteria, Isolation of genomic DNA from Gram-positive bacteria). The PCR amplification was performed using universal fungal primers ITS1 (5′-TCCGTAGGTGAACCTGCGG-3′) and ITS4 (5′-TCCTCCGCTTATTGATATGC-3′) [9]. PCR amplification was carried out on a total volume of 50 µL. The reaction mixture was prepared as follows: 5XGreen GoTaq^®^ Flexi Buffer (Promega, Madison, WI, USA), 2.5 mM of MgCl_2_ (Promega, Madison, WI, USA), 0.2 mM of each dNTP (Global Life Sciences Solutions Operations, Little Chalfont, UK), 0.4 mM of each primer, 1.25 units of GoTaq^®^ Hot Start Polymerase (Promega, Madison, WI, USA), 3 µL of template DNA and Nuclease-Free Water (Thermo Fisher Scientific, Austin, TX, USA). PCR amplification was performed in a Mastercycler Nexus X2 (Eppendorf AG, Hamburg, Germany) set to the following conditions: denaturation at 95 °C for 5 min; 35 cycles of 94 °C for 45 s, 56 °C for 45 s, and 72 °C for 1 min; and a final extension at 72 °C for 10 min. The PCR product was run in a 2% agarose gel containing Midori Green Advance (NIPPON Genetics^®^, Europe GmbH, Düren, Germany).

The specific amplicon of around 700 base pairs (bp) was purified using QIAquick PCR Purification Kit (QIAGEN^®^) according to the manufacturer’s instructions. Quality and quantity of the PCR product were assessed photometrically using a Biophotometer (Eppendorf AG, Hamburg, Germany). Sequencing reactions were carried out in both directions, with the same primers used for PCR amplification, using BrilliantDye^TM^ Terminator v3.1 Cycle Sequencing Kit (NimaGen^®^, Nijmegen, The Netherlands). Sequencing reactions were run in a 3500 Genetic Analyzer (Applied Biosystem, Foster City, CA, USA). The obtained consensus sequence was analyzed using BLAST in GenBank [10]: it showed 100% query cover, 0.0 E-value, and 100.00% identity with *T. indotineae* (Accession No. OQ975444). The newly generated sequence was aligned by using BioEdit v7.2.5 software [11] to check the similarity with reference isolates, including two *T. interdigitale* strains (LC508732 and LC508731) and the *T. indotineae* LC508728 strain [12] to confirm the result. The alignment showed some nucleotide differences between the authors’ sequence and *T. interdigitale* strains, whereas 100% was found to be identical to *T. indotineae* LC508728. Therefore, the strain’s ITS sequence was deposited in the GenBank database under accession number OR192943. 

**Figure 4:**
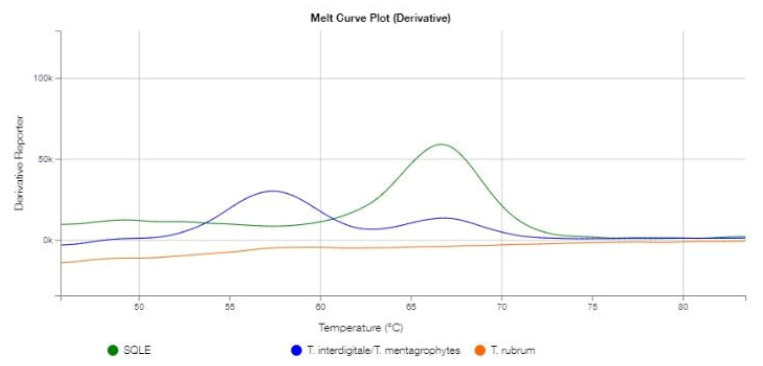
Graphical representation of the melting curve analysis of the analyzed *T. indotineae* strain. The melting peak of dermatophytes species (T_m_ 57.5 °C) is shown in the blue channel. The melting peak of *SQLE* gene (T_m_ 66.8 °C) is shown in the green channel.

Considering that *T. indotineae* is commonly known as a multidrug-resistant dermatophyte, a DermaGenius^®^ Resistance Multiplex real-time PCR assay (Pathonostics^®^, Maastricht, The Netherlands) was carried out to detect the presence of the most common (Phe397Leu and Leu393Phe) along with the less prevalent (Leu393Ser, Phe397Ile, and Phe397Val) *SQLE* gene mutants that confer terbinafine resistance [13]. However, according to the manufacturer’s information, actual differentiation between different mutant strains is not possible. The results of the DermaGenius^®^ PCR assay are shown in Figure 4. 

The blue channel peak (T_m_-value: 57.5 °C) suggests that the analyzed sample belonged to the *T. mentagrophytes*/*T. interdigitale* complex (range 57.0–59.5 °C), which includes *T. indotineae* according to the manufacturer’s information, and no peaks in the orange channel suggest that the strain was not a *T. rubrum*. The green channel peak suggests a specific melting temperature (T_m_-value: 66.8 °C) for the analyzed strain, revealing its terbinafine susceptibility, which falls within the range of temperatures associated with wild-type *SQLE* strains (range 64.5–68.0 °C) [13]. 

Fortunately, in vitro data obtained agreed with the in vivo remission of the clinical manifestation: at the end of the prescribed 12 weeks of treatment, the skin lesions were in complete clinical remission, as well as the nail alterations. 

The dermatologist explained to the patient the scientific relevance of her clinical case and she gave her consent for publication. 

## 3. Discussion

In the present manuscript, the authors described the first clinical case of a sensitive *T. indotineae* strain causing *tinea corporis* and onychomycosis in Central Italy. Firstly, the dermatophyte was identified by its macro- and microscopical features as belonging to the *T. mentagrophytes*/*T. interdigitale* species complex. Subsequently, the PCR and sequencing of the ITS region of ribosomal DNA provided the identification as *T. indotineae*. Lastly, a DermaGenius^®^ Resistance Multiplex real-time PCR assay was carried out to establish terbinafine resistance or susceptibility of the strain. The melting curve analysis classified the strain as terbinafine sensitive as well as the wild-type strain included in the kit as a positive control. Genotypic characterization was in agreement with clinical remission of the *tinea corporis* manifestation after oral terbinafine treatment. Onychomycosis improvement was also observed following topical ciclopirox nail solution. Fortunately, despite intrafamilial transmission of *T. indotineae*, infection seems to be common [3] and the patient has a lively social life in the Indian community; no other relatives or friends have shown similar skin or nails lesions so far. However, it should be emphasized that, despite the repeated invitations to have the cohabitants checked free of charge, none of the patient’s relatives made themselves available for the visit. The epidemiological information was therefore based only on what the patient reported. 

*T. indotineae* has been found in India since 2004 when its first nucleotide sequence, obtained from a human skin sample, was deposited in GenBank (accession number AB430471.1) [14]. In addition, inappropriate use of antibiotics, antifungals, and corticosteroids have increased terbinafine resistance up to 75% compared to the 44% resistance rate for *T. rubrum* [15,16]. Lack of hygiene, overcrowding, work in a hot and humid environment, and wearing tight-fitting synthetic clothing are other potential factors of *T. indotineae* infection in developing countries [17]. Details not to be underestimated could be the intensive migrations and the recovery of tourism post-COVID-19 pandemic that could have spread some pathogens, including *T. indotineae*. Moreover, the anthropophilic pattern of *T. indotineae* could promote an inter-human transmission, so much that familial cases were detected for about 50% of the patients and the sharing of fomites was particularly incriminated [16]. 

Very recently, Bortoluzzi et al. [5] found four other cases of *tinea corporis* and *tinea cruris*, without onychomycosis, caused by *T. indotineae* in Northern Italy differing from the present case because of their terbinafine-resistance. In the authors’ opinion, it seems unusual that despite the relevant immigration to Italy from *T. indotineae*-endemic countries, so few cases caused by this fungus have been reported. Probably, similar cases are not correctly identified. Primarily, in common practice, Italian dermatologists do not perform mycological tests for the dermatophyte identification and limit themselves to a clinical diagnosis when it is clearly evident. Sometimes not even therapy resistance induces the dermatologists to investigate further and, if the lesions do not regress, the antifungal treatment is simply changed. Additionally, conventional methods not supported by molecular techniques are not adequate at differentiating between the *T. mentagrophytes*/*T. interdigitale* complex and the *T. indotineae* strains, which may lead to an underestimation of the latter. 

*Trichophyton indotineae* poses a public health issue due to the uncontrolled situation in India and risk of international spreading [4]. Unfortunately, superficial cutaneous mycoses are not considered pivotal in terms of public health. Generally, health systems pay more attention to mortality than morbidity, underestimating the dermatophyte role in public health. Dermatophytoses are usually considered to be “minor” diseases, which does not take into consideration the impact on the quality of life and the relevance that such infections can assume among fragile populations (young, old, pregnant, immunocompromised—YOPI). The risks become of great importance especially when the antifungal-resistance occurs. The high level of terbinafine resistance of *T. indotineae*, associated to a limited number of antifungal agents available to treat dermatophytosis and an inappropriate prescriptions of them, highlights the importance of a reliable diagnosis [17]. Molecular assays based on PCR and sequencing overcome the conventional methods limitations. Furthermore, the real-time PCR on *SQLE* gene allowed a real estimated data of antifungal resistance of *T. indotineae* isolates to be obtained. 

On May 2023, the European Centre for Disease Prevention and Control (ECDC) reported (source: US CDC’s Morbidity and Mortality Weekly Report—MMWR, 12 May 2023) two cases of severe *tinea* caused by antifungal-resistant *T. indotineae* in the United States, which were never isolated before in the country. ECDC encouraged the use of the EpiPulse to implement rapid information sharing at the European level in case of concerning clusters or cases of *T. indotineae*. Therefore, the Italian Ministry of Health informed all the Regions and Autonomous Provinces accordingly. The case described in this manuscript was reported locally to the Health and Welfare Regional Department (Umbria region), then to the Ministry of Health, which reported the information at the European level through the EpiPulse.

## 4. Conclusions

*Tinea* is highly contagious, causing inflammation and pruritis that can become severe. There is no systematic public health surveillance of severe *tinea* cases. These rare, difficult-to-treat infections can be expected to increase, and awareness among clinicians needs to be raised. *T. indotineae* infection should be considered when lesions occur in patients coming from endemic areas or with travel history linked to these countries. Moreover, widespread *tinea* not improving with topical or oral terbinafine could suggest the involvement of this new dermatophyte species. In particular, specialized laboratories may be required for correct identification via genomic sequencing.

In this manuscript, the first clinical case of *tinea corporis* and onychomycosis due to a terbinafine-sensitive *T. indotineae* strain in Central Italy was reported. To promote active surveillance programs to control dermatophytosis, synergic actions between human dermatologists and veterinarians should be encouraged for the management of this clinical case. *Trichophyton indotineae* detection in Northern and Central Italy and its role in human health are two crucial items that will encourage the authors to further improve diagnostic iter through a One Health approach. 

## Data Availability

The *Trichophyton indotineae* isolate 27025_3_2023 can be found under the accession number OR192943 in the GenBank database.
